# Cytotoxic T Lymphocyte Activation Signals Modulate Cytoskeletal Dynamics and Mechanical Force Generation

**DOI:** 10.3389/fimmu.2022.779888

**Published:** 2022-03-16

**Authors:** Aashli Pathni, Altuğ Özçelikkale, Ivan Rey-Suarez, Lei Li, Scott Davis, Nate Rogers, Zhengguo Xiao, Arpita Upadhyaya

**Affiliations:** ^1^ Biological Sciences Graduate Program, University of Maryland, College Park, MD, United States; ^2^ Institute for Physical Science and Technology, University of Maryland, College Park, MD, United States; ^3^ Department of Mechanical Engineering, Middle East Technical University, Ankara, Turkey; ^4^ Department of Animal and Avian Sciences, University of Maryland, College Park, MD, United States; ^5^ Department of Physics, University of Maryland, College Park, MD, United States

**Keywords:** CD8+ cytotoxic T lymphocyte, cytoskeleton, traction force, IL-12 cytokine, actin, microtubule, lytic granule, myosin

## Abstract

Cytotoxic T lymphocytes (CTLs) play an integral role in the adaptive immune response by killing infected cells. Antigen presenting cells (APCs), such as dendritic cells, present pathogenic peptides to the T cell receptor on the CTL surface and co-stimulatory signals required for complete activation. Activated CTLs secrete lytic granules containing enzymes that trigger target cell death at the CTL-target contact, also known as the immune synapse (IS). The actin and microtubule cytoskeletons are instrumental in the killing of CTL targets. Lytic granules are transported along microtubules to the IS, where granule secretion is facilitated by actin depletion and recovery. Furthermore, actomyosin contractility promotes target cell death by mediating mechanical force exertion at the IS. Recent studies have shown that inflammatory cytokines produced by APCs, such as interleukin-12 (IL-12), act as a third signal for CTL activation and enhance CTL proliferation and effector function. However, the biophysical mechanisms mediating such enhanced effector function remain unclear. We hypothesized that the third signal for CTL activation, IL-12, modulates cytoskeletal dynamics and force exertion at the IS, thus potentiating CTL effector function. Here, we used live cell total internal reflection fluorescence (TIRF) microscopy to study actomyosin and microtubule dynamics at the IS of murine primary CTLs activated in the presence of peptide-MHC and co-stimulation alone (two signals), or additionally with IL-12 (three signals). We found that three signal-activated CTLs have altered actin flows, myosin dynamics and microtubule growth rates as compared to two signal-activated CTLs. We further showed that lytic granules in three-signal activated CTLs are less clustered and have lower velocities than in two-signal activated CTLs. Finally, we used traction force microscopy to show that three signal-activated CTLs exert greater traction forces than two signal-activated CTLs. Our results demonstrate that activation of CTLs in the presence of IL-12 leads to differential modulation of the cytoskeleton, thereby augmenting the mechanical response of CTLs to their targets. This indicates a potential physical mechanism *via* which the third signal can enhance the CTL response.

## Introduction

CD8+ T cells or cytotoxic T lymphocytes (CTLs) play an integral role in the adaptive immune response triggered by pathogens and in tumor surveillance. Antigen presenting cells (APCs) process and present pathogenic peptides bound to class I major histocompatibility complex (MHC) molecules on their cell membranes. The T cell receptor (TCR) on naïve CD8+ T cells is stimulated after recognition of a cognate peptide-MHC complex on the APC membrane. A second co-stimulatory signal is required for complete CTL activation and is provided by binding of B7-1/2 proteins expressed on the APC surface to the CD28 receptor on the naïve T cell membrane (1). Once activated, CTLs recognize and kill infected or damaged host cells by secreting lytic granules containing perforins and granzymes, which collectively act to promote target cell apoptosis.

Inflammatory cytokines, such as interleukin-12 (IL-12) and interferons α and β (IFN-α/β), have been recently characterized as a third signal for T cell activation. *In vitro* studies have shown that IL-12 is able to drive naïve CD8+ T cells to differentiate into potent effector CTLs (1). CD8+ T cells activated with peptide-MHC and co-stimulatory proteins in the presence of IL-12 (**three signal**, or **3SI**) exhibit increased IL-2 signaling and enhanced proliferation as compared to CD8+ T cells activated in the presence of peptide-MHC and co-stimulation alone (**two signal**, or **2SI**) (1–4). While both 2SI and 3SI CTLs produce effector proteins and are able to lyse target cells, 3SI CTLs have increased expression of granzymes and perforin, contributing to increased cytolytic efficiency both *in vitro* and *in vivo* (2–8). Target cell killing by 3SI CTLs is also potentially augmented by increased efficiency of synapse formation as compared to 2SI CTLs (9). While the above studies have used population-scale experiments such as flow cytometry and microarray analysis to study the enhancement of effector function in 3SI CTLs, the mechanisms by which the third signal may modulate cytotoxic efficiency have not been explored at the cellular scale.

At the single-cell level, the T cell cytoskeleton is intricately involved during activation and cytolytic function at the CTL-target synapse, also known as the immunological synapse (IS) (10). Following initial contact of a CTL with a target cell, TCR engagement triggers a downstream signaling cascade which results in dramatic cytoskeletal rearrangements beginning with initial actin polymerization at the contact site (11–14). The cytoskeletal motor proteins dynein and myosin II act in tandem to promote translocation of the centrosome towards the IS (15–18). Centrosome translocation is followed by docking at the synapse, which is required for the transport of lytic granules along microtubules towards the target cell (19, 20). The microtubule motors dynein and kinesin facilitate granule transport towards the minus or plus end of microtubules respectively, aiding in the approach of lytic granules towards the contact site (19, 21, 22). An increase in intracellular calcium flux following TCR activation is also required for granule transport and release (23–25).

Actin dynamics regulate secretion of lytic granules at the IS: within the first minute of contact with the target cell, actin rapidly depletes across the center of the IS, allowing lytic granules to dock and fuse with the CTL plasma membrane. Granule secretion is halted within a few minutes by the recovery of cortical actin, which acts as a physical barrier to granule release (14, 26, 27). Additionally, T cells exert mechanical forces during activation and IS formation (28–34). These forces are known to be actomyosin and microtubule-dependent (30, 32–34). Force exertion by CTLs is spatiotemporally correlated with lytic granule secretion at the IS and potentiates the efficacy of target cell killing by creating local hotspots of increased membrane tension, which promotes pore formation by the effector protein perforin (31).

The current paradigm for the role of the cytoskeleton in CTL function has been informed by studies performed in CTLs activated in the absence of the third signal. Recent work has shown that IL-12 treatment modulates the expression of cytoskeletal regulators such as Rho GTPases and Rho-GTPase-activating proteins (Rho-GAPs) in CD4+ and CD8+ T cells (4, 35–37). Furthermore, IL-12 treatment activates central signaling pathways that regulate actin depletion and recovery at the IS (4, 8, 26, 27, 38) and are required for cytoskeletal force generation in CTLs (29, 31). Together, these studies suggest that activation of CD8+ T cells in the presence of IL-12 may enhance CTL function by modulating cytoskeletal dynamics and forces, suggesting a mechanochemical pathway that links cytokine stimulation to CTL effector function.

Here, we examine the role for the third signal for CTL activation, IL-12, in modulating cytoskeletal dynamics and mechanical force generation at the IS. We use high resolution microscopy to study cytoskeletal dynamics and force generation in murine CTLs activated in the presence and absence of IL-12. Our data show that CTL activation in the presence of the third signal results in altered cytoskeletal repatterning at the CTL-target interface, leading to a larger actin depletion zone, slower actin speeds and faster microtubule growth rates. We also find differences in lytic granule dynamics with three-signal activated CTLs exhibiting a higher number of lytic granules and slower granule speeds. Finally, using traction force microscopy (TFM), we show that three-signal activated CTLs generate higher traction forces at the IS. Thus, by regulating the cytoskeleton, cytokine stimulation of CTLs may provide a potential mechanism for augmenting their killing response during the immune response.

## Materials and Methods

### Plasmids

pEGFP-C1 F-tractin-EGFP was a gift from Dyche Mullins (Addgene plasmid #58473). Lamp1-RFP was a gift from Walther Mothes (Addgene plasmid #1817). The EGFP-EB3 and MLC-EGFP plasmids were gifts from Dr. Robert Fischer, National Heart, Lung, and Blood Institute.

### Purification of Naïve OT-I CD8+ T Cells

Isolation of naïve OT-I CD8+ T cells was performed as described before (39). Briefly, peripheral lymph nodes were collected from euthanized OT-I mice and homogenized in 15 mL glass grinders (DWK Life Sciences, Millville, NJ), to become single cell suspension. The cells were washed with complete Allos medium, followed by filtering through a 70 µm filter (VWR, Radnor, PA). Cells were incubated with FITC-labeled antibodies specific to B220, CD4, CD44, CD11c, and I-Ab (BioLegend, San Diego, CA) for 25 minutes at 4°C. After washing with Allos, the resuspended cells were incubated for 20 minutes at 4°C with anti-FITC conjugated magnetic MicroBeads (Miltenyi Biotech, Auburn CA). The cells were washed and passed through separation columns inserted in a MACS magnet. Cells that flowed through the columns were collected, which were > 94% CD8+ and <0.5% CD25. Allos medium is RPMI-1640 supplemented with fetal calf serum (10%), HEPES (10 mM), MEM non-essential amino acid (1×), sodium pyruvate (1 mM), penicillin and streptomycin (100 U/mL), and 2-mercaptoethanol (50 μM) (Mediatech, Manassas, VA).

### Activation of Naïve OT-I CD8+ T Cells by 2SI and 3SI

Flat-bottom microtiter wells in 24-well plates were coated with Dimer X-2Kb:Ig fusion protein loaded with OVA257–264 peptide (BD Pharmingen, San Jose, CA) and recombinant B7-1/Fc chimeric protein (R&D Systems, Minneapolis, MN) as previously described (42). For two signal (2SI) stimulation, purified naive OT-I CD8+ T cells were plated at a density of 3 × 10^5^ cells in 1.5 mL Allos medium in each well of a coated plate with 2.5 U/mL recombinant human IL-2 (R&D Systems, Minneapolis, MN). For three signal (3SI) stimulation, naive OT-I CD8 T cells were plated under 2SI conditions as described above and additionally supplemented with 2 U/mL of murine rIL-12 (R&D Systems, Minneapolis, MN). The cells were incubated for three days before further analysis.

### Killing Assay

The CellTiter-Glo^®^ (CTG) killing assay is based on the number of viable cells left in the culture after cytotoxic T lymphocyte killing of the target cells (39, 40). B16.OVA melanoma cells adhere to plastic surfaces and can efficiently present OVA_257–264_ peptide; activated OT-I T cells recognize H-2K^b^/OVA_257–264_ and initiate specific killing of these B16.OVA cells (41, 42). B16.OVA cells were seeded onto 96-well white plates at 30,000 cells/well in 100 μL Allos medium, and activated OT-I cells were added to each well as effectors to target cells (B16.OVA cells) at a ratio of 1:1, 5:1, and 20:1. After overnight incubation, T cell suspensions (both OT-I cells and B16.OVA) were removed by washing three times with Allos medium. Luminescent signals (relative luminescent unit, RLU) from a 96-well plate were measured by the addition of 200 μL of 50% Cell Titer Glo (Promega, Madison, WI) followed by measurement of luminesce using a plate reader (Bio-Rad). The kill percentage of the B16.OVA cells by effector OT-I cells was calculated according to the following equation: Killed % = 100% x (RLU of untreated B16.OVA cells – RLU of B16.OVA cells cultured with OT-I cells)/RLU of untreated B16.OVA.

### Cell Culture and Transient Transfections

The Neon electroporation system (Invitrogen, Waltham, MA) was used for transient transfection of activated CTLs. Transfections were performed two days prior to the experiment according to the following protocol: 1.5-2 × 10^5^ cells were resuspended in 10 μL of R buffer with 0.5-2 μg of plasmid. The cells were electroporated under the following conditions: 1325V/10ms/3 pulses. Transfected cells were transferred to fresh pre-warmed Allos medium and incubated at 37°C with 5% CO_2_ for 36-48 hours prior to imaging.

### Substrate Preparation

8-well chambers (Cellvis, Mountain View, CA) were incubated with 0.01% poly-L-lysine (PLL) diluted in distilled water for 10 mins at room temperature. PLL was aspirated from each well and the chambers were allowed to dry for 1 hour at 37°C. PLL-coated dishes were subsequently incubated with a 10 μg/mL solution of purified anti-mouse CD3 antibody (Clone 17A2, BioLegend, San Diego, CA) in 1x Dulbecco’s phosphate-buffered saline (DPBS) for 2 hours at 37°C or overnight at 4°C. The coated wells were washed with Leibovitz’s L-15 (L-15) imaging medium prior to the experiment.

### Immunofluorescence

Activated CTLs were stimulated on anti-CD3 coated coverslips and fixed at 3 and 6 minutes after stimulation using 3.5% paraformaldehyde for 10 minutes. Fixed cells were washed thoroughly with 1X DPBS. Cells were permeabilized for 8 minutes with a 0.15% Triton-X solution. Blocking was performed using 0.02g/mL bovine serum albumin (BSA) and 0.3M Glycine in 1X DPBS solution for 1 hour at room temperature. Acti-stain 488 phalloidin (Cytoskeleton, Inc., Denver, CO) was used to label F-actin according to the manufacturer’s instructions.

### Total Internal Reflection Fluorescence (TIRF) Microscopy

Imaging was performed on an inverted microscope (Nikon Ti-E PFS, Melville NY) equipped with a 60x objective for IRM and TFM, and a 100× objective lens TIRF imaging respectively using a Prime BSI camera (Photometrics, Tucson AZ). Imaging protocols were implemented using Nikon Elements and images were cropped in Fiji before further analysis using MATLAB scripts. For live cell imaging, activated CTLs in L-15 medium were seeded on anti-CD3 coated surfaces equilibrated to 37°C in a stage-top Okolab Incubator (Okolab S. R. L., Pozzuoli, NA, Italy). IRM time-lapse images were acquired every 5 s. TIRF images were acquired every 0.5-1 s for imaging of F-Tractin-EGFP transfected cells and every 1 s for imaging of EGFP-EB3 transfected cells.

Fast imaging of lytic granules: Rapid TIRF imaging of lytic granules was performed using a 100x objective and an electron multiplying charge coupled device (emCCD) camera (Andor iXon 897). Activated CTLs expressing Lamp1-RFP were imaged between 1 to 15 minutes after being added to an anti-CD3-coated coverslip. For each cell, timelapse images were acquired every 100 ms for 100 s (1000 frames).

### Traction Force Microscopy

Coverslip activation, polyacrylamide gel fabrication and gel surface functionalization were performed as described previously (43). Briefly, 35 mm glass bottom dishes with No. 1.5 coverslips (Cellvis, Mountain View, CA) were activated by incubating with 2% 3-aminopropyltrimethoxysilane (Sigma-Aldrich, St. Louis, MO) followed by thorough washing, drying and incubation with 1% glutaraldehyde (Fisher Scientific, Hampton, NH). Dried coverslips were used for fabrication of two-layer polyacrylamide gels with a thin layer of fluorescent beads on top. A ratio of 3% acrylamide to 0.1% bis-acrylamide was used for preparation of polyacrylamide gels with individual gel stiffnesses in the range of 0.7-1.2 kPa. Indentation by stainless steel spheres was used to quantify gel stiffness as before (30). Prepared polyacrylamide gels were functionalized using hydrazine hydrate and coated with 0.01% poly-L-lysine. Functionalized gels were washed with 1x DPBS and incubated with 10 μg/mL purified anti-mouse CD3 antibody for 2 hours at 37°C or overnight at 4°C, followed by washing with 1xDPBS. PBS was replaced with pre-warmed L-15 medium prior to imaging.

Activated CTLs were added to anti-CD3 coated polyacrylamide gels. The cells were allowed to adhere to the substrate and imaging was initiated within 5 minutes of addition of cells. Images of CTLs in brightfield and the fluorescent bead field were acquired every 15 s for a total of 15 minutes using widefield microscopy. CTLs were detached from the substrate using 2 mL each of 0.25% trypsin-EDTA and 1x DPBS. A final image of the fluorescent bead field was acquired after detachment of CTLs to be used as a reference frame.

For inhibitor treatments, activated CTLs were added to anti-CD3 coated polyacrylamide gels and were imaged as above until 15 minutes after addition of cells. Y27632 was added to the imaging medium at a final concentration of 100 μM, and imaging was performed for another 15 minutes. CTLs were detached using trypsin-EDTA and 1x DPBS as before, and a final reference image of the fluorescent bead field was captured.

### Image Analysis

#### Area Calculation From IRM Images

Edge detection using the Canny operator in MATLAB was implemented to segment IRM images to obtain a binary mask of the cell. The area over time curves were fit to a hyperbolic tangent function *A*(*t*) ∼ *A*_0_ tanh*(αt)* to obtain the cell spreading rate *α*.

#### Intensity Analysis of Fixed Cells

Edge detection was implemented as above to segment images of phalloidin-labeled CTLs. The binary mask of the cell was eroded with a disk of radius 0.3*radius of the cell to define a ‘central’ and an ‘annular’ region. The ratio of total actin intensity in the cell center to total actin intensity in the annulus was calculated to quantify actin depletion at the cell center. The Wilcoxon rank sum test was used to test the null hypothesis of actin intensity ratios being derived from the same population.

#### Spatio-Temporal Image Correlation Spectroscopy (STICS)

STICS analysis (44, 45) was run to generate actin and myosin flow vector maps after the cell had achieved maximum spread area for up to five minutes after the start of imaging. STICS was run with a subregion size of 16 x 16 pixels with a shift of 4 pixels between subregions. For analysis of actin flows, the time of interest (TOI) window was selected to be 20 seconds, with a TOI shift of 2 seconds. For analysis of myosin flows, the time of interest (TOI) window was selected to be 60 seconds, with a TOI shift of 6 seconds. Directionality was defined as the cosine of the angle between a given actin flow vector and a vector from the same region of the cell pointing toward the cell center. All vectors with directionality greater than 0.9 were defined as inward flows. The two-sample Kolmogorov Smirnov test was used to test the null hypothesis of actin flow speeds originating from the same distribution. The proportions test was used to test the null hypothesis of the two proportions of inward flow being identical.

#### Tracking of EB3 Tips

Analysis of EB3 tips was performed after the cell had achieved maximum spread area for up to five minutes after the start of imaging. EB3 tips were tracked using the comet detection routine of the MATLAB-based software u-track (46). A Brownian search radius of 1 to 6 pixels (corresponding to 0.06-0.39 µm) was used for frame-to-frame linking.

#### Analysis of Granule Distribution

To quantify granule distribution four frames (30 seconds apart) from the live imaging of Lamp1-RFP cells were chosen to detect and measure bright spots corresponding to individual granules or granule clumps. The cell edge was detected from the maximum intensity projection of the live cell movie. The individual frames were binarized and granules were segmented using the function *imbinarize* from MATLAB. Thresholding values used for each image were defined based on the mean of the distribution of pixel intensities in an automated manner to account for photobleaching and differences in Lamp1-RFP expression. Detected objects smaller than 3 pixels (~ 0.5 µm) were discarded and granule area fraction was calculated as the ratio of number of pixels within the granule and the total number of pixels in the cell.

#### Tracking of Lytic Granules

Individual lytic granules were detected and tracked using the Fiji plugin TrackMate (47). The following parameters were used for detection and tracking of lytic granules: estimated blob diameter = 1 µm, threshold = 0.5, maximum linking distance = 0.6 µm, maximum gap closing distance = 0.6 µm, maximum gap closing frames = 2. Tracks with a duration lower than 2 seconds were excluded from further analysis. MSD curves were calculated for each track using the MATLAB per-value class @msdanalyzer (48). A one-degree polynomial fit was performed for the log-log of the MSD curves to obtain a value of *α* for each curve. Only tracks with fits with an r-squared value greater than 0.8 were retained for further analysis.

#### Traction Force Analysis

Microscopy images were registered using ImageJ/Fiji (descriptor-based registration) and cropped into 600 x 600 pixel-sized regions of interests (ROIs) each containing a single cell at the center. Traction force microscopy analysis was conducted on the single-cell ROIs based on Regularized Fourier Transform Traction Cytometry (FTTC) (49) using a open-source package implemented in MATLAB (50). The displacement field was estimated using a template matching algorithm with a template size of 32 pixels and maximum displacement of 16 pixels. The values for elastic modulus and gel height were obtained by indentation experiments mentioned above. Regularization parameter for stress estimation was 0.0001. Calculated stress fields at each time point were integrated over the entire ROI and reported as traction force applied by each cell. Traction force metrics including the maximum, mean and median force during the 15-minute imaging period were considered for comparison of 2SI and 3SI CTLs. For inhibitor experiments on 3SI CTL, the effect of treatment was quantified by the ratio of traction force at the end of treatment to the traction force at the onset of treatment for each cell. Statistical comparisons of traction force and force ratio were based on Wilcoxon rank sum test.

## Results

### CTL Activation Conditions Influence the Size and Organization of the Immune Synapse

TCR triggering by APCs leads to rapid cytoskeletal reorganization at the cell-cell interface, which is essential for proper CTL function (14). Cytoskeletal remodeling leads to the rapid spreading of CTLs on the target cell or on stimulatory surfaces (11). We sought to investigate how the presence of inflammatory cytokines as a third activating signal impacts CD8+ T cell cytoskeletal dynamics upon TCR restimulation. We purified naïve CD8+ T cells from lymph nodes of OT-I mice (**Figure S1A**) and activated these *in vitro* under 2SI or 3SI conditions. We verified that 3SI activated cells expressed increased levels of CD25 and similar CD62L levels as compared to 2SI activated cells (**Figure S1B**). Next, we confirmed the functional activity of 2SI and 3SI CTLs by measuring effector molecule production and cytolytic efficiency. Consistent with the typical phenotype of effectors generated by 2SI and 3SI, we found that 3SI CTLs exhibited increased IFN-γ and granzyme B production, accompanied by enhanced cytolytic efficiency (**Figures S1C, D**) (3, 4, 51).

**Figure 1:**
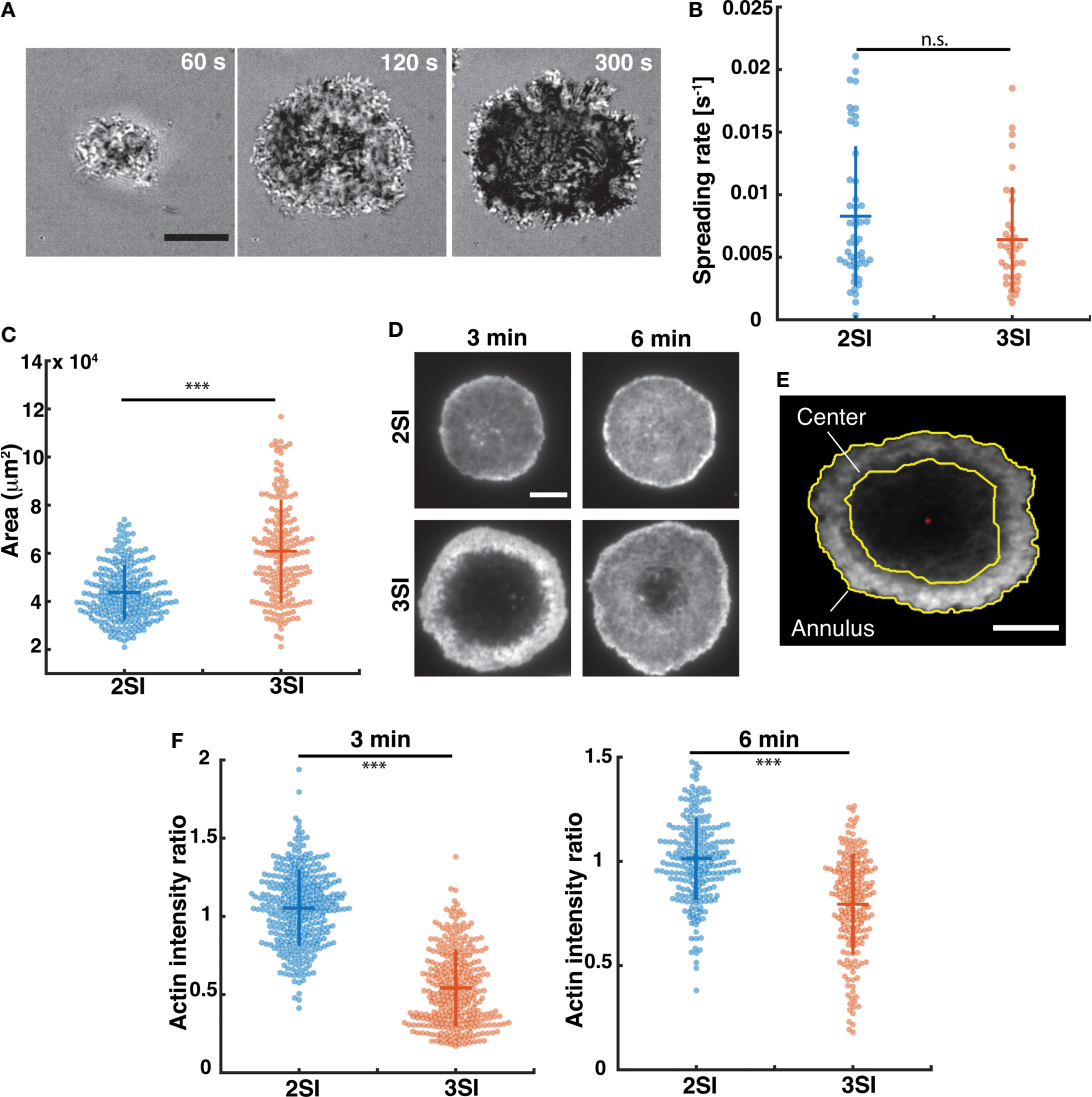
Spreading kinetics and actin distribution in activated CTLs. **(A)** Representative time-lapse images of a 3SI CTL spreading on an anti-CD3-coated coverslip and imaged using IRM. Scale bar = 10 µm. **(B)** Spreading rates of 2SI (n=48 cells), 3SI (n=36 cells) CTLs from at least 3 independent experiments. **(C)** Representative TIRF images of 2SI and 3SI CTLs fixed at 3 and 6 minutes and stained with phalloidin to visualize F-actin. **(D)** Immune synapse area obtained from TIRF images of phalloidin-stained 2SI and 3SI CTLs fixed at 6 min. **(E)** Depiction of the erosion of a binary mask to define central and annular regions in the cell. Red asterisk indicates the cell centroid. **(F)** Actin intensity ratio at 3 and 6 min. Actin intensity ratio was defined at the ratio of actin MFI at the center to the actin MFI at the annulus. Scale bar = 5 um for panels **(D, E)**. Data taken from at least 150 cells from 3 independent experiments. Wilcoxon rank-sum test performed to calculate p-values. ***p<0.001, n.s. - not significant.

To visualize the contact interface, activated CTLs were imaged during interaction with an anti-CD3-coated stimulatory glass coverslip using interference reflection microscopy (IRM) (**Figures 1A** and **S2A**). Upon contact with the coverslip, cells started spreading on the surface, forming an extended contact zone or immune synapse. To compare spreading dynamics between 2SI and 3SI CTLs, the cell area was calculated using segmented images (see Methods). The change in area over time was fit to a hyperbolic tangent function, which has been previously used to describe T cell spreading (52, 53). Fitted curves were used to calculate the rate of cell spreading (**Figure S2B**). We find that 2SI and 3SI CTLs have comparable spreading rates (**Figure 1B**). We further measured the contact area from images of CTLs fixed at 6 minutes, a timepoint at which most CTLs achieved the maximum spread area in our experiments, and found that 3SI CTLs display a larger IS area than 2SI CTLs (**Figure 1C**).

TCR stimulation on anti-CD3 coated surfaces in activated CTLs induces rapid actin depletion at the immune synapse, followed by actin recovery (14, 26). This depletion of cortical actin at immune synapse is required for secretion of lytic granules and release of granule contents towards the target cell (14, 26). To study the formation and recovery of this actin ‘ring’, we next compared the organization of the actin cytoskeleton in 2SI and 3SI CTLs. Activated 2SI and 3SI CTLs stimulated on anti-CD3-coated glass coverslips were fixed at 3 and 6 minutes after stimulation to capture ‘early’ and ‘late’ stages of actin reorganization at the IS. To study F-actin distribution, cells were stained with phalloidin and imaged using TIRF microscopy (**Figure 1D**). Qualitatively, we observed a larger actin depletion zone in 3SI cells, with this effect enhanced at early timepoints (**Figure 1D**). From the fluorescent images, we defined two regions – the cell ‘center’ and ‘annulus’ as shown in **Figure 1E**. The mean fluorescence intensity (MFI), which measures the total fluorescence intensity in a given region divided by the area of the region, was calculated for these two regions. The ratio of actin MFI at the cell center to actin MFI in the annulus (henceforth referred to as the ‘actin intensity ratio’) was compared across activation conditions. An actin intensity ratio equal to 1 would signify a relatively homogenous distribution of actin at the synapse, while a ratio greater than or lesser than 1 could arise due to central actin accumulation or depletion respectively. We found that 3SI CTLs consistently displayed enhanced actin depletion at the center of the IS as compared to 2SI CTLs at both early and late timepoints. (**Figure 1F**). Importantly, the observed difference in actin intensity ratio is due to reduced actin MFI at the center of 3SI CTLs at both 3 and 6 minutes (**Figure S2C**).

### 3SI CTLs Exhibit Altered Lytic Granule Distribution and Dynamics as Compared to 2SI CTLs

Activated CTLs kill target cells by secreting lytic granules containing perforin and granzymes that trigger target cell apoptosis. Lytic granule secretion occurs in zones of actin depletion in both CTLs and natural killer cells (14, 26, 54, 55). Furthermore, lytic granule movement in natural killer cells is modulated by the cytoskeleton – inhibiting various elements of the cytoskeleton using small molecular inhibitors of actin or microtubules leads to altered granule mobility patterns (56, 57). In CTLs, depolymerization of actin using Latrunculin A leads to continued granule release (26). Given that the cytoskeleton regulates lytic granule dynamics, enhanced actin depletion in 3SI CTLs may be associated with altered granule dynamics at the interface, potentially resulting in increased lytic granule secretion as compared to 2SI CTLs. We therefore studied lytic granule dynamics in 2SI and 3SI activated CTLs co-transfected with F-Tractin-EGFP and Lamp1-RFP (Lysosomal-associated membrane protein 1) to label F-actin and lytic granules respectively. While Lamp1 is a lysosomal membrane marker, previous studies have shown that over 90% of Lamp1-labeled lysosomes in CTLs contain cathepsin-D and granzymes found in lytic granules (58). Lamp1 tagging has also been previously used to track lytic granule mobility and release in CTLs and natural killer cells (14, 26, 55, 58). Transfected CTLs were stimulated on anti-CD3 coated glass coverslips and imaged with TIRF every 1 second (**Figure S3A**). In agreement with previous work, both 2SI and 3SI CTLs show characteristic actin ring formation and recovery, with granule secretion occurring in areas of the synapse where actin is transiently depleted (**Figures S3B, C**).

**Figure 2:**
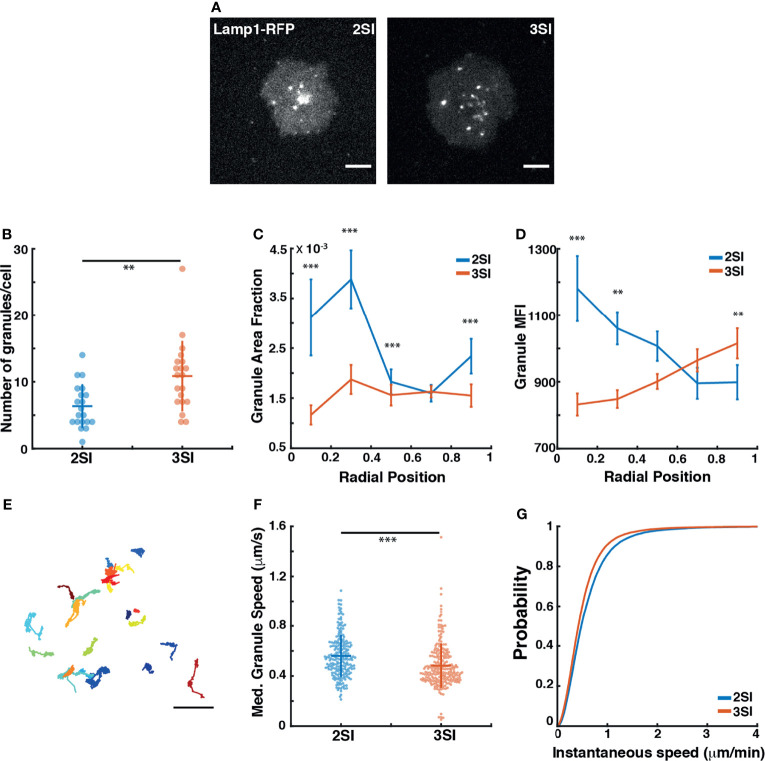
3SI CTLs exhibit altered lytic granule distribution and dynamics: **(A)** Representative snapshots of a 2SI and 3SI CTL expressing Lamp1-RFP interacting with an anti-CD3-coated coverslip imaged using TIRF microscopy after reaching maximum spread area. Scale bar = 5 µm. **(B)** Number of granules for 2SI and 3SI CTLs detected per cell using thresholding. For granule number, *p*=0.002 (Wilcoxon rank-sum test). **(C)** Fraction of the complete cell area occupied by lytic granules granules and **(D)** Granule MFI binned according to granule radial position. Data is presented as mean ± SEM. **(E)** Full lytic tracks for a representative 3SI CTL. Scale bar = 2 µm. **(F)** Median granule speeds and **(G)** cumulative distribution function of instantaneous granule speeds obtained from tracking of lytic granules. For median granule speeds, p=6.762793e-08 (Wilcoxon rank-sum test). For instantaneous granule speeds, p=0.005 (two-sample Kolmogorov-Smirnov test). Data taken from 20 cells each for 2SI (n=205 tracks) and 3SI (n=226 tracks) from 2 independent experiments. ***p<0.001, **p<0.01.

To capture lytic granule dynamics near the plasma membrane prior to release, we performed rapid TIRF imaging of activated CTLs transfected with Lamp1-RFP at intervals of 100 ms (**Supplementary Movie 1**). Fast imaging of Lamp1-RFP-labeled lytic granules showed a variety of mobility patterns, with a larger fraction exhibiting low mobility, while others displayed fast directed motion – likely the result of transport along microtubules. Qualitatively, 3SI CTLs appeared to have a greater number of lytic granules at the synapse than 2SI CTLs. Furthermore, lytic granules in 2SI CTLs appeared to be more clustered towards the center of the synapse, while 3SI CTLs displayed a relatively homogenous distribution of granules across the synapse (**Figure 2A**). To quantify these differences, we used thresholding to detect individual granule-sized objects in the captured images. Consistent with our qualitative observations, a higher number of structures were detected per cell for 3SI CTLs than for 2SI CTLs (**Figure 2B**). From the detected structures, we calculated the mean granule fluorescence intensity and granule area as a fraction of the cell area. This data was then summarized in five bins based on radial position of the granule from the cell center, where a radial position of 0 corresponds to the cell centroid. We found that for 2SI CTLs, granules tend to occupy a larger fraction of the cell area closer to the cell center (corresponding to radial position bins 0-0.2 and 0.2-0.4) (**Figure 2C**). In 3SI CTLs, granules were distributed more evenly across the synapse compared to the more clustered appearance in 2SI cells. Furthermore, granules closer to the cell center in 2SI CTLs tended to have a higher MFI than in 3SI, reflecting a high degree of granule accumulation at the center of the synapse (**Figure 2D**).

We then tracked granule movements at the synapse (**Figure 2E**) and calculated instantaneous and median granule speeds. We found that 3SI CTLs exhibit significantly slower granule speeds across the synapse as compared to 2SI CTLs (**Figures 2F, G**). Finally, the mean-squared displacement (MSD) was calculated for each track to capture the degree of overall granule movement. The first 25% of each MSD curve was fit according to the relationship *MSD*(*t*) *= Bt^α^* to obtain a value of α for each track (48, 59). The resulting α values for each track were used to classify the track as exhibiting super-diffusion (α > 1.2), normal diffusion (1.2 > α > 0.8) or sub-diffusion (α < 0.8). 2SI and 3SI CTLs both show similar fractions of super-diffusive, normal, and sub-diffusive tracks (**Figure S3E**). Classified tracks were also used to construct ensemble MSDs for both 2SI and 3SI CTLs, with the resultant ensemble MSD curves showing similar behavior regardless of activation condition (**Figure S3D**). In summary, these results show that, while activation conditions do not influence overall lytic granule mobility patterns, 3SI CTLs exhibit altered granule distribution and speeds as compared to 2SI CTLs.

### Cytokine Stimulation Alters Actomyosin Dynamics

**Figure 3:**
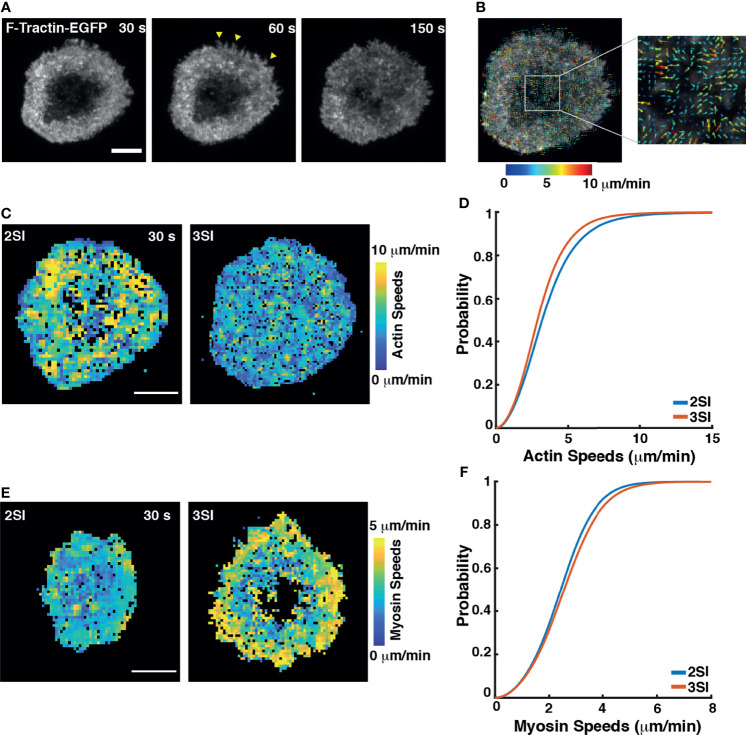
3SI stimulation alters actomyosin flow speeds in CTLs: **(A)** Representative time-lapse images of a 3SI CTL expressing F-tractin-EGFP spreading on an anti-CD3-coated coverslip and imaged using TIRF microscopy. Yellow arrows show actin protrusions. **(B)** Output from STICS analysis showing actin flow vectors at the immune synapse. The color of actin flow vectors corresponds to the magnitude of actin speeds, as represented by the color bar. **(C)** Heat maps showing magnitudes of actin flow speeds over the synapse at the indicated timepoint for 2SI and 3SI CTLs. Colors correspond to speeds as indicated by the color bar. **(D)** Cumulative distribution function of actin flow speeds obtained from STICS analysis of 2SI and 3SI CTLs. n=15 cells for 2SI and 12 cells for 3SI from 6 independent experiments. For actin speeds, p= 8.09e-06, two sample Kolmogorov-Smirnov test. Scale bars = 5 µm. **(E)** Heat maps showing myosin flow speeds at the indicated time point in 2SI and 3SI cells. **(F)** Cumulative distribution function of myosin flow speeds obtained from STICS analysis of 2SI and 3SI CTLs. n=11 cells for 2SI and 17 cells for 3SI from 3 independent experiments. For myosin speeds, p=8e-04, two sample Kolmogorov-Smirnov test. Scale bars = 5 µm.

Actin clearance at the center of the immune synapse is accompanied by extensive actin flow at the periphery of the synapse. Actin flows drive the centripetal movement of TCR signaling microclusters towards the center of the synapse and trigger downstream signaling resulting in sustained calcium flux (11, 60–66). Having observed differences in actin organization at the IS in 2SI and 3SI CTLs, we next investigated actin flow dynamics using high-resolution live-cell imaging methods. We transfected activated CTLs with F-Tractin-EGFP to label F-actin. Transfected CTLs were stimulated on anti-CD3 coated glass coverslips as described above and imaged using TIRF microscopy every 1 second (**Figure 3A** and **Figure S4A**, **Supplementary Movie 2**). Live TIRF imaging of activated CTLs expressing F-Tractin-EGFP revealed dynamic protrusions and retractions at the lamellipodial edge. Both 2SI and 3SI CTLs exhibited transient actin depletion at the center of the synapse, followed by recovery of cortical actin (as seen in the last panel of **Figure 3A** and **Figure S3A**) which coincides with termination of granule fusion (26).

Actin flows during the first 5 minutes after the cell had achieved its maximum spread area were analyzed using Spatio-Temporal Image Correlation Spectroscopy (STICS). STICS is a technique that quantifies spatiotemporal changes in protein dynamics based on fluorescence intensity correlations across spatial and temporal interrogation windows (44, 45). A representative output image depicting actin flow velocity vectors color-coded for magnitude overlaid on an image of an activated CTL is shown in **Figure 3B**. We decomposed actin flow velocity vectors into heatmaps of speed magnitudes and directionalities (**Figures 3C** and **S4B**). Actin flow directionality was defined as the cosine of the angle between an actin flow vector and a vector from the same region of the cell pointing towards the cell center.

To quantify differences in actin flows between 2SI and 3SI CTLs, we constructed the cumulative distribution function (CDF) of actin speeds calculated using STICS. We found that 3SI CTLs exhibited significantly slower actin flow speeds at the synapse as compared to 2SI CTLs (**Figures 3D** and **S4C**). We further constructed probability density functions (PDFs) of actin flow directions to compare directionalities between conditions. All vectors with directionality greater than 0.9 were classified as ‘inward flows’. Our analysis showed that while directionalities for 2SI and 3SI CTLs followed a similar distribution, 3SI CTLs had significantly higher inward flows (**Figure S4D**).

High-resolution imaging of the IS has implicated myosin II in forming arc-like structures at the base of the peripheral regions of the contact zone (60) that were shown to drive the inward movement of engaged TCR clusters. We next examined whether cytokine stimulation alters myosin dynamics. We transfected activated CTLs with EGFP-tagged myosin light chain (MLC) to label myosin II. Transfected CTLs were stimulated on anti-CD3 coated glass coverslips as described above and imaged using TIRF microscopy every 1 second (**Supplementary Movie 3**). STICS analysis of myosin II dynamics revealed that 3SI stimulated cells showed more coherent and higher overall myosin speeds compared to 2SI stimulated cells (**Figure S4E**, **Figures 3E, F**).

### 3SI CTLs Display Faster Microtubule Growth Rates As Compared To 2SI CTLs

**Figure 4:**
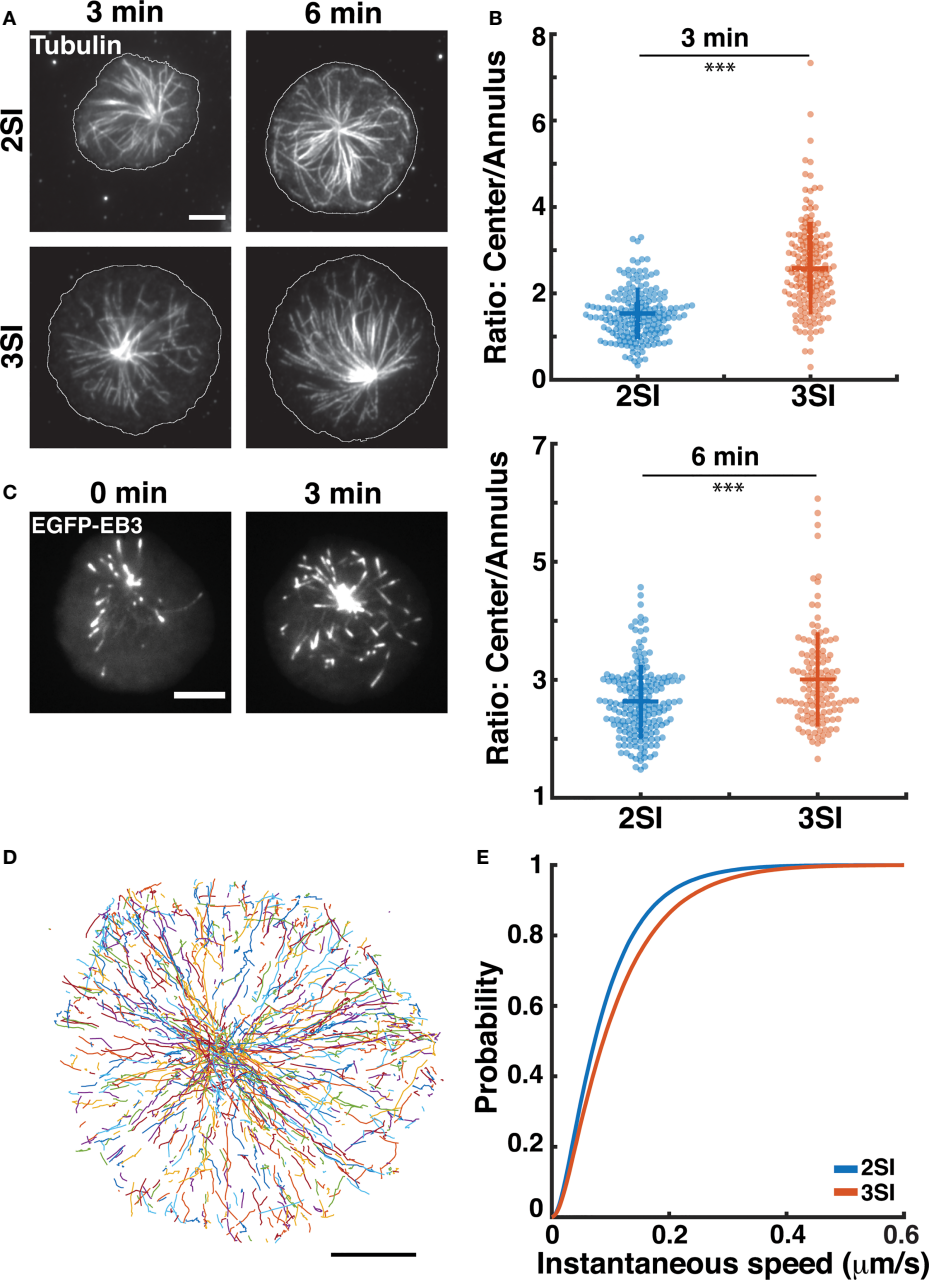
3SI CTLs display altered microtubule growth rates: **(A)** Representative TIRF images of 2SI and 3SI CTLs fixed at 3 and 6 min and stained with anti-β-tubulin to visualize microtubules. Cell outline is shown in white. **(B)** Ratio of tubulin fluorescence intensity at the center to the annulus at 3 and 6 min. Data taken from at least 130 cells from 3 independent experiments. Wilcoxon rank-sum test performed to calculate p-values. ***p<0.001. **(C)** Representative time-lapse images of a 3SI CTL expressing EGFP-EB3 spreading on an anti-CD3-coated coverslip and imaged using TIRF microscopy. **(D)** All EB3 tracks for a representative 3SI cell. **(E)** Cumulative distribution function of instantaneous microtubule tip. n=12 cells for 2SI, 16 cells for 3SI from 4 independent experiments. For EB3 speeds, p=0.009 (two sample Kolmogorov-Smirnov test). All scale bars represent 5 µm.

Contact formation between CTLs and target cells induces centrosome reorientation towards the IS with lytic granules being transported along microtubules (MTs) to the CTL-target cell synapse (19, 20). Our prior work has demonstrated that actin flow patterns at the IS further influence MT dynamics at the IS (32, 67). Having observed differences in lytic granule distribution and actin flows, we decided to investigate whether the activation of CTLs in the presence of inflammatory cytokines would also affect MT distribution and dynamics. We first used immunofluorescence to study microtubule organization at the IS of 2SI and 3SI CTLs. Activated CTLs stimulated on anti-CD3-coated glass coverslips were fixed at 3 and 6 minutes after stimulation, similar to the methodology used to study actin distribution. Fixed cells were stained with antibodies to tubulin and imaged using TIRF microscopy (**Figure 4A**). Comparison of tubulin MFI in central and annular regions of the cell revealed that 3SI CTLs had a higher ratio of tubulin intensity at the cell center to annulus at both timepoints (**Figure 4B**). This effect arises due to a lower MFI of tubulin intensity at the annulus of 3SI CTLs, likely caused by a higher accumulation of actin in the same region (**Figures S5B** and **Figures 1D**, **3A**).

To study microtubule growth rates, we transfected activated CTLs with EGFP-EB3, a construct that labels EB3 (end-binding protein 3). EB3 binds to the ends of growing microtubules and is frequently used to study microtubule growth (68). Transfected CTLs were stimulated on anti-CD3 coated glass coverslips as above and imaged using TIRF at 1 second intervals (**Figure 4C** and **Figure S5A**, **Supplementary Movie 4**). We tracked EB3 comets during the first 5 minutes after the cell had achieved its maximum spread area using the MATLAB-based software, uTrack (46) (**Figure 4D**). The instantaneous speed of a microtubule tip was defined as the inter-frame tip displacement between two consecutive images divided by the imaging interval (1 second) (67). We constructed the CDF of instantaneous EB3 speeds to compare the distributions of microtubule tip speeds between activation conditions and found that 3SI CTLs exhibited significantly faster EB3 speeds than 2SI CTLs (**Figure 4E**). These results indicate faster microtubule growth rates in 3SI CTLs as compared to 2SI CTLs.

### Traction Force Microscopy Reveals 3SI CTLs Generate Stronger Traction Forces Than 2SI CTLs

The extensive actin flows and MT dynamics during the formation of the IS mediate force generation at the CTL-target contact site. These forces enhance target cell killing, potentially by altering the membrane and cytoskeletal tension of the target cell, thereby facilitating perforin activity and target cell lysis (30–33). Given our above results on differences in actin and microtubule dynamics in 3SI CTLs and to further investigate the role of the third signal in modulating cytoskeletal dynamics, we proceeded to examine mechanical force generation by activated CTLs.

**Figure 5:**
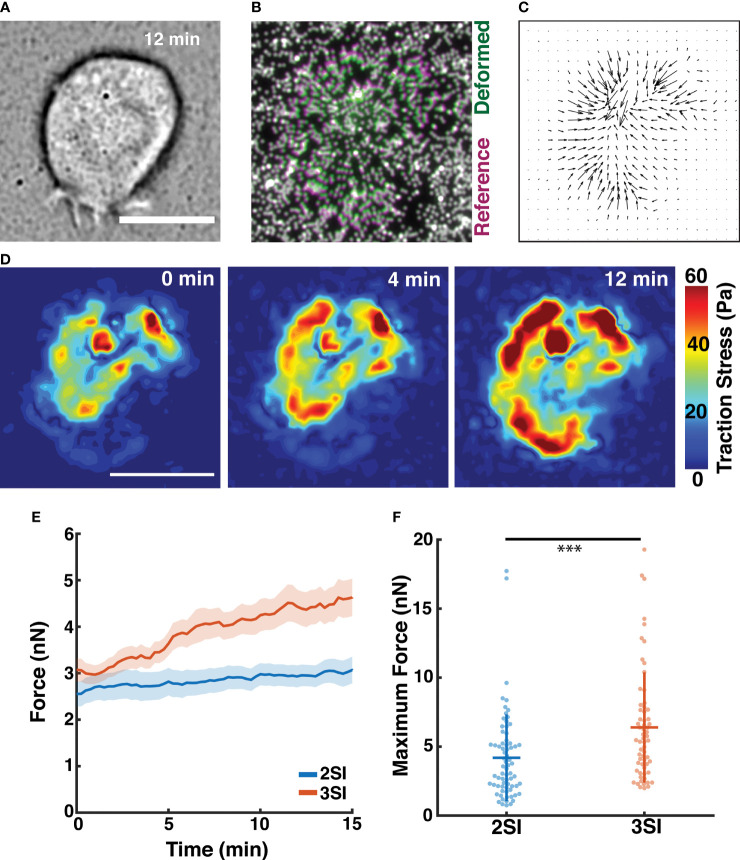
Traction force microscopy reveals 3SI CTLs generate stronger traction forces. **(A)** Brightfield image of a 3SI CTL spreading on a polyacrylamide hydrogel substrate at 12 minutes of imaging. **(B)** Composite fluorescence image showing displacement of fluorescent beads embedded in the gel surface during spreading of the cell in **(A)**. Bead positions for deformed and undeformed (reference) states of the hydrogel are indicated by green and magenta, respectively. Undisplaced beads are indicated by white. **(C)** Traction stress vectors calculated for the cell in **(A)**. **(D)** Spatial distribution of traction stress magnitude associated with the cell in **(A)** at time points between 0 and 12 minutes of imaging. **(E)** Evolution of integrated traction force over the course of imaging for 2SI and 3SI CTLs. The trends are reported as mean ± sem. **(F)** Maximum traction force generated by 2SI and 3SI cells during the imaging period. n = 72 cells for 2SI and 63 cells for 3SI. Data taken from 4 independent experiments. *** p-value < 0.0001. All scale bars indicate 10 μm.

We used traction force microscopy (TFM) to measure forces generated by 2SI and 3SI CTLs during stimulation by a biomimetic stimulatory surface. We prepared polyacrylamide (PA) hydrogel substrates of approximately 1.0 kPa stiffness embedded with fluorescent nanoparticles and coated with anti-CD3. The chosen stiffness level for the hydrogel substrates matches the range of stiffness reported for various cancer cell lines and was considered appropriate for modeling the target cell (69–71). Activated CTLs were plated on PA gels and the fluorescent nanoparticle field was imaged every 15 seconds using widefield microscopy during the interaction of CTLs with the gel surface (**Figure 5A** and **Supplementary Movie 5**). A reference image of the nanoparticle field was obtained by detaching the cells with trypsin-EDTA and washing the gel with PBS after the end of imaging. Force exertion by CTLs on the PA hydrogel substrate causes displacement of fluorescent nanoparticles. Displacement fields were obtained for each cell by comparing the locations of fluorescent nanoparticles with respect to the reference image (**Figure 5B**). The measured displacements were used to calculate the traction stresses exerted by the cell based on the linear elastic theory for a homogenous isotropic material of known stiffness and thickness (**Figure 5C**, **Methods**).

2SI and 3SI CTLs rapidly begin to exert a force after contact with the stimulatory PA gel building up to saturating levels (~10-15 min, **Figure 5D** and **Supplementary Figure S6A**). Enhanced traction stresses were observed in an annular region at the cell periphery, likely corresponding to the actomyosin ring. The baseline level of force at 0 min resulted from a finite time lag between addition of cells on the substrate and the start of imaging. The total forces exerted by primary CTLs were in the nano-newton (nN) range, comparable to previously measured values (31, 33) (**Figure 5E**). Intriguingly, our results show that 3SI CTLs exert significantly greater traction forces than 2SI CTLs (**Figures 5E, F** and **Figure S6B**). Previous work has shown that myosin II is required for force generation in T cells (30, 31). Analogous to previous observations, we found that force exertion by 3SI CTLs was reduced after treatment with the Rho-kinase inhibitor Y27632 which reduces myosin II activity (72) (**Figure S6C**). Observed differences in force generation between 2SI and 3SI CTLs are potentially indicative of differential modulation of the cytoskeletal machinery by third signal cytokines such as IL-12.

## Discussion

Optimal function of effector CD8+ T cells requires integration of multiple signals, including cytokine stimulation as a third signal (73). A number of studies have led to the well-established paradigm that third signal cytokines such as IL-12 induce the optimal accumulation of activated CD8+ T cell populations *in vivo* by regulating their division and survival (74) or by conferring a proliferative advantage (38, 51, 75). In addition, prior exposure to third signal cytokines leads to rewiring of TCR signaling pathways (76) and increased expression of perforin and granzymes (3, 4), potentially mediating their enhanced effector function.

In this work, we used a combination of high resolution TIRF imaging and traction force microscopy to delineate an additional mechanism by which third signal cytokines enhance effector function by regulation of the CTL cytoskeleton. We showed that 3SI CTLs exhibit higher actin depletion as compared to 2SI CTLs. This is accompanied by larger numbers of lytic granules and decreased clustering of lytic granules towards the center of the synapse in 3SI CTLs. Given that actin depletion regulates secretion of lytic granules in CD8^+^ T cells (14, 26), these results likely indicate an improved ability of 3SI CTLs to secrete lytic granules, hence contributing to enhanced cytotoxic efficiency.

We further found that activation in the presence of IL-12 leads to changes in cytoskeletal dynamics, with 3SI CTLs exhibiting slower actin flows compared to 2SI CTLs. Intriguingly, we found that myosin speeds are higher in 3SI CTLs at the synaptic interface. Actin retrograde flow is a result of the ‘superimposition’ of two processes - actin assembly and myosin-based filament retraction (77, 78). Previous studies have found that myosin-II has a larger influence on flows in primary T cells, where flows are mostly localized to the lamellar region of the cell (79). Of note, we observed that activated CTLs show coherent myosin retrograde flow whereas inward actin flow is not as organized.

Actomyosin dynamics are known to drive cellular force generation in T cells. Our results showed that 3SI CTLs exert higher traction forces than 2SI CTLs with higher myosin-II speeds being an important contributor toward the exertion of traction forces in CD8+ T cells. Overall, our observations that slower actin flow rates in the distal region of the IS are correlated with higher traction forces are broadly consistent with prior work on adherent cells (80). These differences in mechanical force exertion between 2SI and 3SI CTLs have important functional implications. Basu et al. have shown that mechanical force exertion across the CTL-target interface facilitates perforin pore formation, thereby suggesting a correlation between actomyosin generated forces and cytotoxic potential (31). The enhanced force generation by 3SI CTLs may provide an additional mechanism by which cytokines augment effector function.

A central question that remains is the mechanism by which the actin cytoskeleton may be differentially regulated in 3SI CTLs. Previous work comparing gene expression in 2SI and 3SI CTLs at timepoints similar to those used in our study may shed light on potential molecular players (4). Primary among these are the Rho-family GTPases RhoA, Rac and Cdc42. 3SI CTLs exhibit lower expression of GTPase-activating proteins Rho-GAP1 and Rac-GAP1 which participate in GTPase ‘molecular switching’ by triggering GTPase behavior of Rho, leading to GTP dissociation and conversion to an ‘off’ state. This is consistent with our results showing increased myosin speeds and cellular forces which would be expected with enhanced Rho activity. Additionally, IL-12 treatment results in decreased expression of Cdc42 and its effector proteins in CD4+ and CD8+ T cells (35), which could lead to the decreased actin flows that we observed. Finally, expression of the Cdc42 effector protein IQGAP1 is differentially modulated in 3SI CTLs. IQGAP1 links microtubule plus ends with the actin cortex and is cleared along with actin at the time of granule secretion delivery (20).

Another important cytoskeletal regulatory pathway that may be involved is *via* phosphatidylinositol-3-kinase (PI3K). IL-12 treatment activates the PI3K signaling pathway in CD8+ T cells (8, 38) and in CD4+ T cells (81, 82). Class IA PI3K isoforms generate the second messenger PIP3 which controls actin ring formation (37). PI3K regulatory subunit p85alpha, which is expressed at higher levels in 3SI CTLs, is recruited to TCR microclusters (4, 37). PI3K signaling is also required for force exertion and IS formation: inhibition of PI3K antagonist PTEN leads to higher forces (31), and an increase in IS size and killing efficiency (37). Examining the connection between IL-12 regulation of the PI3K pathway and enhanced effector function of CTLs will be the subject of future work.

We further found that 3SI CTLs have a higher density of microtubules at the cell center. One possible mechanism regulating reorganization of the MT cytoskeleton in 3SI CTLs may involve differential diacylglycerol (DAG) concentrations. DAG accumulates in the cSMAC and is required for centrosome docking at the IS. Diacylglycerol kinase-*α* (DGK-*α*) localizes to the dSMAC and is required to focus DAG in the cSMAC for proper centrosome polarization (83). DGK-*α* is expressed at lower levels in 3SI CTLs which may result in differential DAG concentrations across the synapse, thus modulating centrosome polarization and reorganization of the MT cytoskeleton (4). We also found that 3SI CTLs have faster microtubule growth rates. It has been previously reported that actin acts as a physical barrier for MT growth (84). Actomyosin dynamics further regulate MT filament curvature and dynamics (67, 85). Furthermore, we have previously shown that actin retrograde flow in T cells is negatively correlated with MT tip influx (32). Thus, faster MT tip speeds in 3SI CTLs may serve to slow actin retrograde flow and augment force generation. These results also underscore the importance of exploring the roles played by different proteins involved in actin-microtubule crosstalk such as IQGAP1 (86), CLIP-170 (87) and Apc (88).

We note that the experimental system used in our studies did not include ICAM-1, the ligand for the T cell integrin LFA-1. LFA-1 localizes to the peripheral supramolecular activation cluster - an annular region of the IS located at an intermediate distance between the cell center and the cell edge (89). The interaction of LFA-1 with ICAM-1 mediates adhesion of a CTL to a target cell, and is required in addition to antigenic stimulation for granule secretion and IS formation on planar lipid bilayers (37, 90–92), LFA-1 engagement further regulates actin assembly and flows (93–96). While anti-CD3 acts as a strong stimulatory signal to induce cytoskeletal reorganization, whether additional LFA-1 engagement would alter the differential cytoskeletal remodeling patterns observed in our study remains an open question.

The cytoskeleton acts as a dynamic framework for the transmission of mechanochemical signals to regulate T cell signaling and gene expression. At the receptor level, the cytoskeleton is intricately linked to the TCR (13, 93), which has been characterized as a mechanosensor (97–101). Not only can forces activate signaling *via* the TCR, piconewton-level actomyosin forces applied through the TCR allow for the formation of catch bonds with cognate ligands and thus participate in peptide discrimination (100–104). At the cellular level, the cytoskeleton allows the T cell to sample mechanical properties, such as stiffness, of the antigen presenting surface, and modulate force generation (30). While our current work involved CTL activation and force measurements on a hydrogel of physiologically relevant stiffness, an important open question is whether three-signal activation conditions prime CD8+ T cells to better recognize and respond to a range of substrate stiffnesses, thus augmenting T cell function.

In conclusion, the results from our current study indicate that three-signal activation conditions modulate cytoskeletal dynamics and forces in CD8+ T cells. Given that the cytoskeleton is an established regulator of T cell signaling and effector function, differential modulation of the cytoskeletal machinery may be a potential mechanism by which IL-12 enhances CTL effector function. Our results provide novel biophysical insights into the role of IL-12 in improving T cell function at the single cell level.

## Data Availability Statement

The raw data supporting the conclusions of this article will be made available by the authors, without undue reservation.

## Ethics Statement

The animal study was reviewed and approved by Institutional Animal Care and Use Committee, University of Maryland, College Park.

## Author Contributions

AP, AO, and AU designed the experiments with input from ZX. AP and AO performed the experiments. AP, AO, and IR-S analyzed the data with help from SD. NR, IR-S, and AU provided guidance on data analysis. LL performed the purification and characterization of naïve and activated murine CD8+ T cells. AP and AU wrote the manuscript, with input from AO, IR-S, and ZX. AU supervised the project. All authors contributed to the article and approved the submitted version.

## Funding

AU and ZX acknowledge support from the grant NIH R01 GM131054. AU acknowledges support from NSF PHY 1806903. AP’s contribution to this research was supported in part by NSF award DGE-1632976. IR-S would like to acknowledge support from the Fulbright-Colciencias scholarship.

## Conflict of Interest

The authors declare that the research was conducted in the absence of any commercial or financial relationships that could be construed as a potential conflict of interest.

## Publisher’s Note

All claims expressed in this article are solely those of the authors and do not necessarily represent those of their affiliated organizations, or those of the publisher, the editors and the reviewers. Any product that may be evaluated in this article, or claim that may be made by its manufacturer, is not guaranteed or endorsed by the publisher.
