# Supplementary material for: Cytotoxic T Lymphocyte Activation Signals Modulate Cytoskeletal Dynamics and Mechanical Force Generation
Source: Front Immunol. 2022 Mar 16;13:779888. doi: 10.3389/fimmu.2022.779888 (PMC8966475; doi:10.3389/fimmu.2022.779888)
Supplement: Supplementary file 1 [file DataSheet_1.pdf]

Supplementary Material

1. Supplementary Movie Captions

Supplementary Movie 1: Time-lapse movies of a 2SI (left) and 3SI (right) CTL transfected with Lamp1-RFP and imaged every 100 ms using TIRF microscopy during interaction with an anti-CD3-coated glass coverslip. Scale bar is 5 µm.

Supplementary Movie 2: Time-lapse movies of a 2SI (left) and 3SI (right) CTL transfected with F-Tractin-EGFP and imaged using TIRF microscopy during interaction with an anti-CD3-coated glass coverslip. Scale bar is 5 µm.

Supplementary Movie 3: Time-lapse movies of a 2SI (left) and 3SI (right) CTL transfected with MLC-EGFP and imaged using TIRF microscopy during interaction with an anti-CD3-coated glass coverslip. Scale bar is 5 µm.

Supplementary Movie 4: Time-lapse movies of a 2SI (left) and 3SI (right) CTL transfected with EGFP-EB3 and imaged using TIRF microscopy during interaction with an anti-CD3-coated glass coverslip. Scale bar is 5 µm.

Supplementary Movie 5: Time-lapse movie of a 3SI CTL (brightfield, left) interacting with a polyacrylamide gel substrate embedded with fluorescent beads (right) during a typical TFM experiment. Scale bar is 10 µm.

2. Supplementary Figures

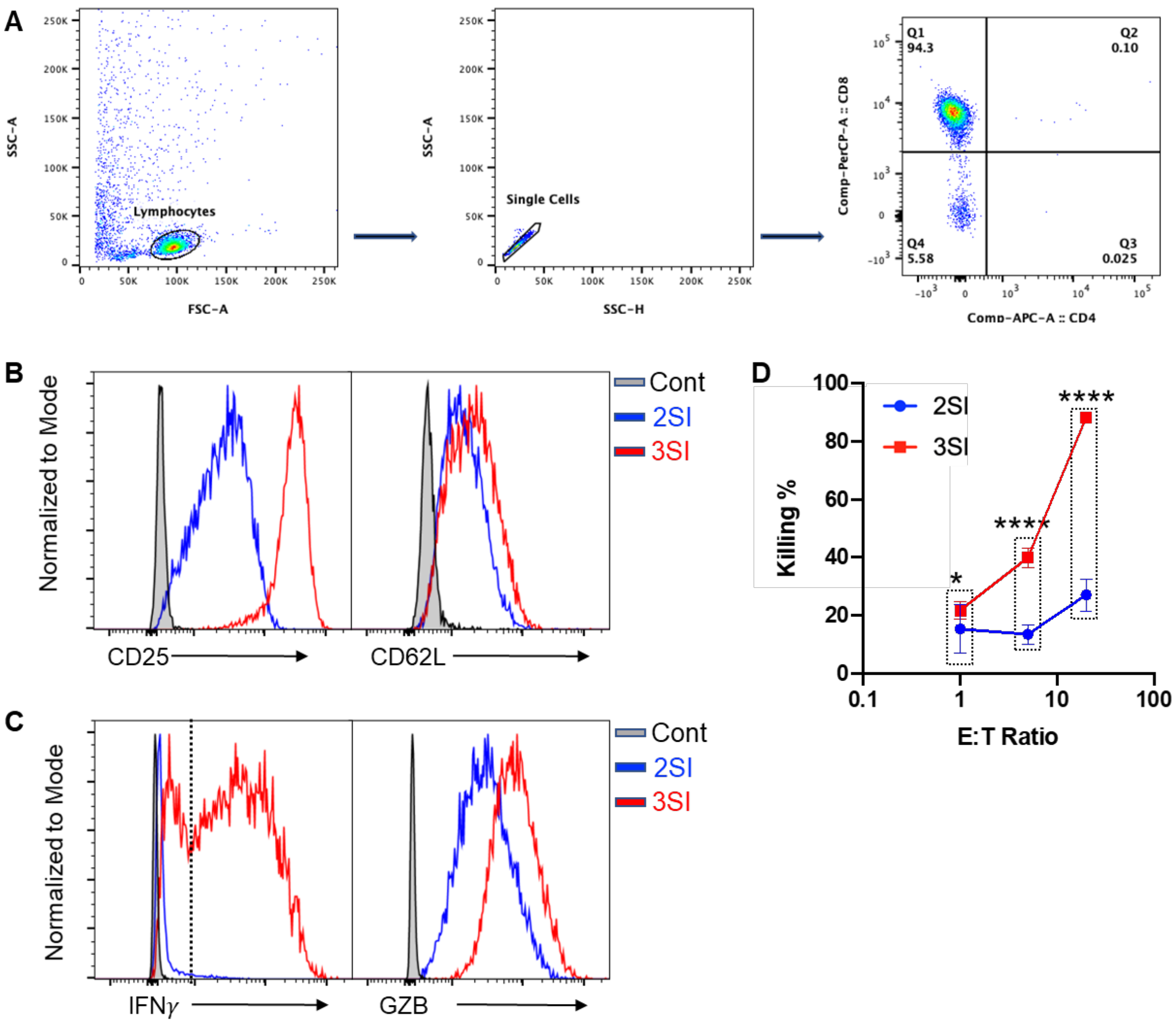

Supplementary Figure 1: Activation of naïve OT-I CD8+ T cells by 2SI and 3SI. Purified naïve OT-I CD8+ T cells were stimulated with 2SI or 3SI for three days. (A) Representative purity check of the negative selected naïve OT-I CD8+ T cells. (B) Comparison of surface expression of CD25 and CD62L between 2SI and 3SI stimulated cells. (C) Comparison of intracellular expression of IFNγ and granzyme B (GZB) between 2SI and 3SI stimulated cells. Control: Isotype staining control. (D) Comparison of killing ability of 2SI and 3SI CTLs on day 3. At day 3, activated 2SI or 3SI effector cells were seeded with B16.OVA at three ratios of E:T (**E**ffectors : **T**argets): 1:1, 5:1 and 20:1. Killed % = 100% x (RLU of untreated B16.OVA cells – RLU of B16.OVA cells cultured with OT-I cells)/RLU of untreated B16.OVA. *$P < 0.05$; ****$P < 0.0001$ by unpaired, two-tailed Student's *t*-test.

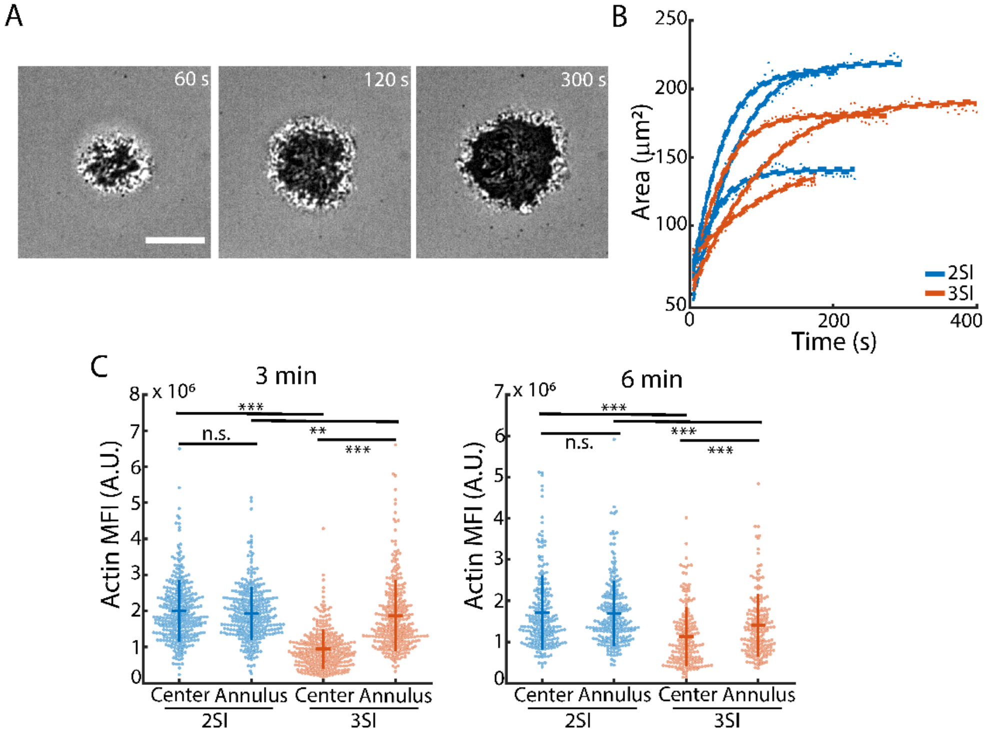

Supplementary Figure 2: Spreading kinetics and actin distribution in activated CTLs: (A) Representative time-lapse images of a 2SI CTL spreading on an anti-CD3-coated coverslip and imaged using IRM. Scale bar = 10 μm. (B) Representative spreading curves for 2SI (blue) and 3SI (red) cells. Data points are shown as dots and tanh fits are shown as smooth curves. (C) Representative TIRF images of 2SI and 3SI CTLs fixed at 3 and 6 minutes and stained with phalloidin to visualize F-actin. (C) Actin MFI at the center and annulus of 2SI (blue) and 3SI (red) CTLs at 3 min (left) and 6 min (right). Data taken from at least 150 cells from 3 independent experiments. Wilcoxon rank-sum test performed to calculate p-values. ***$p<0.001$, **$p<0.01$.

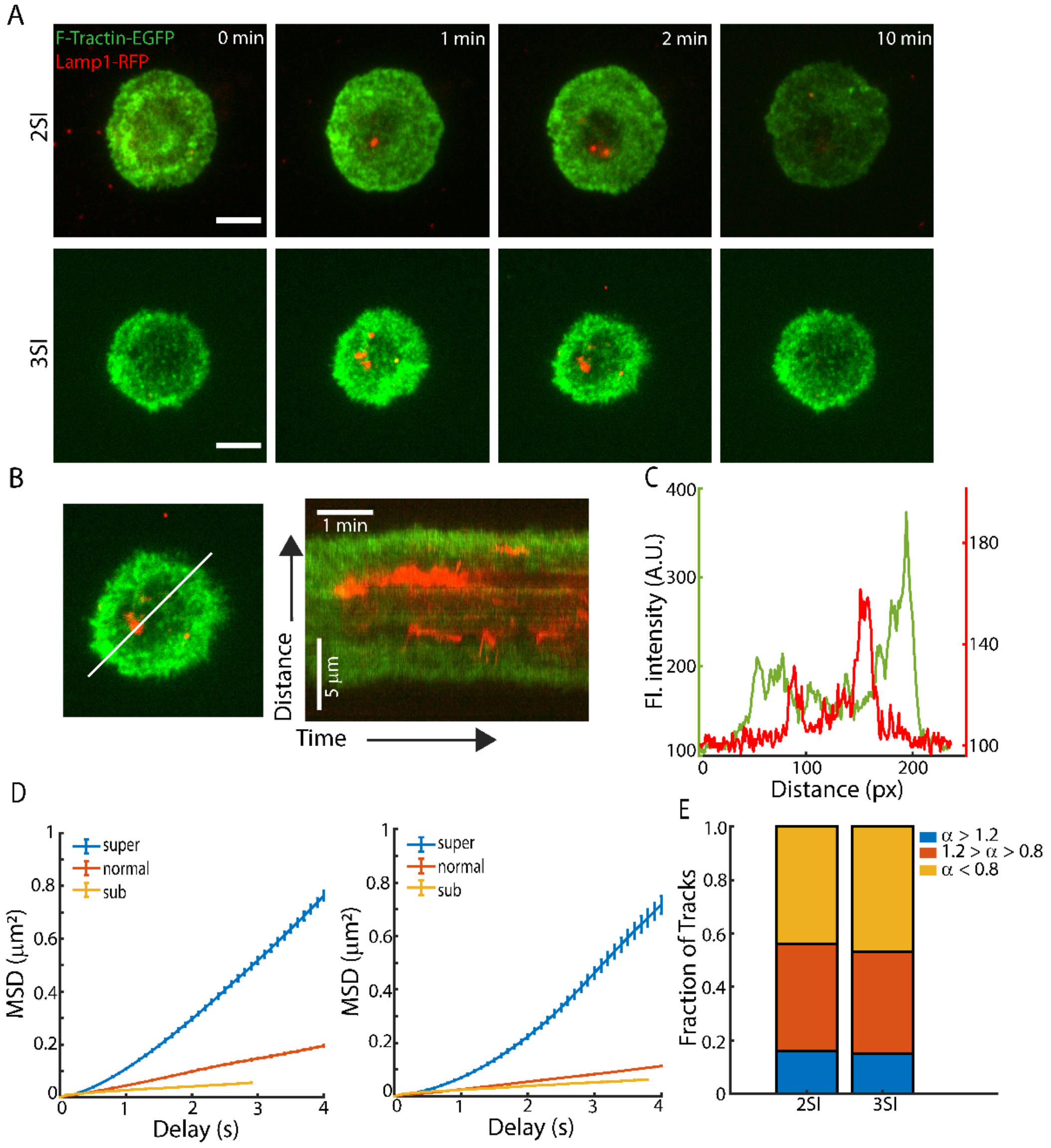

Supplementary Figure 3: Granule dynamics in activated CTLs: (A) Representative time-lapse images of a 2SI and 3SI CTL expressing F-Tractin-EGFP and Lamp1-RFP spreading on an anti-CD3-coated coverslip and imaged using TIRF microscopy. Scale bars = 5 µm. (B and C) Kymograph and fluorescent intensity line profile generated along the indicated axis for a 3SI CTL. (D) Ensemble MSDs for (left) 2SI and (right) 3SI CTLs tracks classified according to α values: super (α > 1.2), normal (1.2 > α > 0.8) and sub (α < 0.8) diffusion. Data is mean ± SEM. (E) Fraction of tracks with α>1.2 (super-diffusive), 1.2>α>0.8 (normal) and α<0.8 (sub-diffusive) for 2SI and 3SI CTLs.

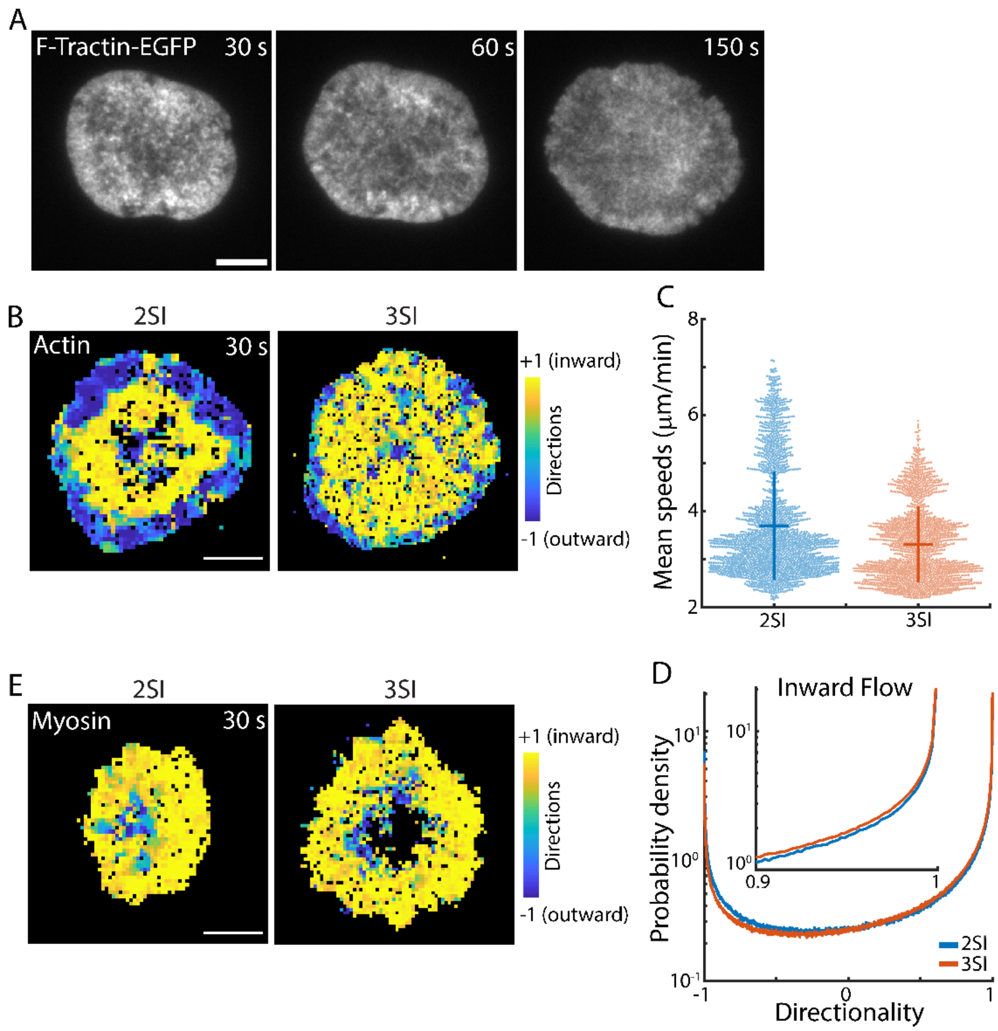

Supplementary Figure 4: 3SI stimulation alters actomyosin flows in CTLs: (A) Representative time-lapse images of a 2SI CTL expressing F-tractin-EGFP spreading on an anti-CD3-coated coverslip and imaged using TIRF microscopy. (B) Spatial heat maps showing directionality of actin flows at the indicated timepoint for 2SI and 3SI CTLs. Colors correspond to directionality as indicated by the color bar. (C) Mean actin flow speeds obtained from STICS analysis of 2SI and 3SI CTLs. (D) The probability density function of actin flow directionalities obtained from STICS analysis of 2SI and 3SI CTLS. (Inset) Flow fractions defined as inward flow (directionality > 0.9). n=15 cells for 2SI, 12 cells for 3SI from 6 independent experiments. For inward flow, p=0.004, Proportions test. (E) Spatial heat maps showing directionality of myosin flows at the indicated timepoint for 2SI and 3SI CTLs. Colors correspond to directionality as indicated by the color bar. All scale bars indicate 5 μm.

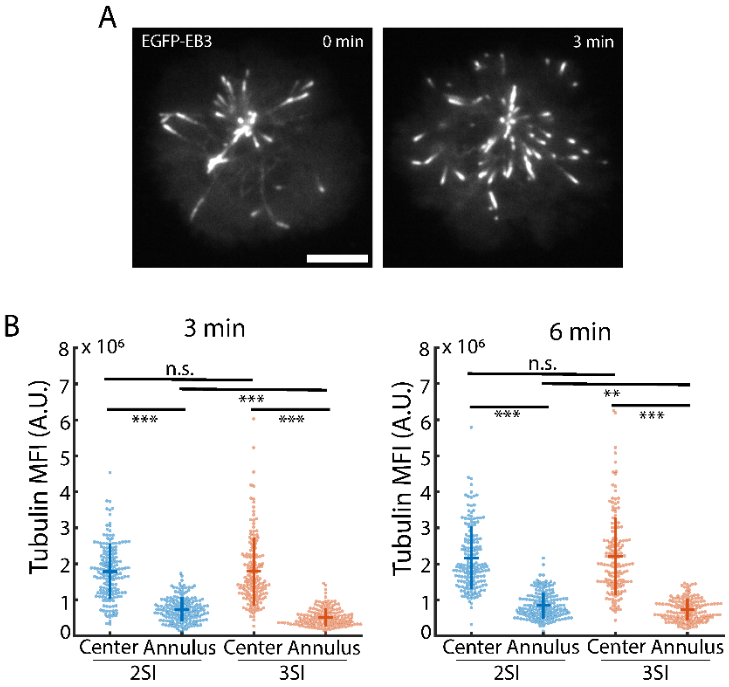

Supplementary Figure 5: 3SI CTLs display altered microtubule organization and growth rates: (A) Representative time-lapse images of a 2SI CTL expressing EGFP-EB3 spreading on an anti-CD3-coated coverslip and imaged using TIRF microscopy. Scale bar = 5 µm. (B) Tubulin MFI at the center and annulus of 2SI (blue) and 3SI (red) CTLs at 3 min (left) and 6 min (right). Data taken from at least 130 cells from 3 independent experiments. ***p<0.001, **p<0.01.

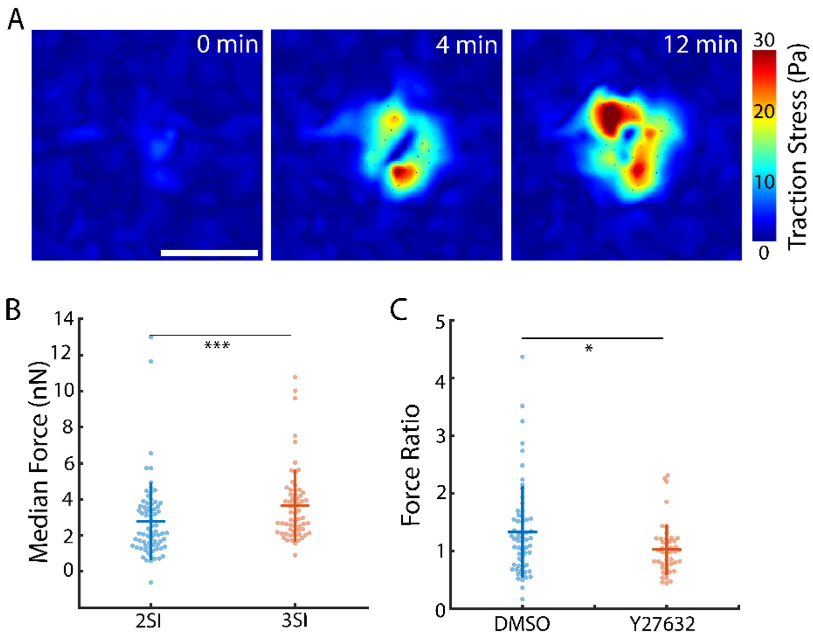

Supplementary Figure 6: Traction force microscopy analysis of 2SI and 3SI CTLs. (A) Spatial distribution of traction stress magnitude associated with a 2SI CTL at time points between 0 and 12 minutes of imaging. Scale bar= 10 µm. (B) Median traction force generated by 2SI and 3SI cells during the imaging period. n = 72 cells for 2SI and 63 cells for 3SI. Data taken from 4 independent experiments. (C) Force ratio for 3SI CTLs treated with DMSO and Y27632. Force ratio is defined as the ratio of force exerted in the last minute of imaging after addition of treatment to the force exerted in the last minute of imaging prior to addition of treatment. n = 61 cells for DMSO control and 46 cells for Y27632 treatment. Data taken from 3 independent experiments. *** $p < 0.0001$; *$p < 0.05$.
